# A study on a broadband photodetector based on hybrid 2D copper oxide/reduced graphene oxide†

**DOI:** 10.1039/d3na00796k

**Published:** 2024-01-25

**Authors:** Duc Anh Ngo, Nhat Minh Nguyen, Cong Khanh Tran, Thi Thanh Van Tran, Nhu Hoa Thi Tran, Thi Thu Thao Bui, Le Thai Duy, Vinh Quang Dang

**Affiliations:** a Faculty of Materials Science and Technology, University of Science 227 Nguyen Van Cu Street District 5 Ho Chi Minh City 700000 Vietnam vinhquangntmk@gmail.com; b Center for Innovative Materials and Architectures (INOMAR) Ho Chi Minh City 700000 Vietnam; c Vietnam National University (VNU-HCM) Ho Chi Minh City 700000 Vietnam

## Abstract

These days, photodetectors are a crucial part of optoelectronic devices, ranging from environmental monitoring to international communication systems. Therefore, fabricating these devices at a low cost but obtaining high sensitivity in a wide range of wavelengths is of great interest. This report introduces a simple solution-processed hybrid 2D structure of CuO and rGO for broadband photodetector applications. Particularly, 2D CuO acts as the active material, absorbing light to generate electron–hole pairs, while 2D rGO plays the role of a transport layer, driving charge carriers between two electrodes. Our device exhibits remarkable sensitivity to a wide wavelength range from 395 nm to 945 nm (vis-NIR region). Interestingly, our devices' responsivity and photoconductive gain were calculated (under 395 nm wavelength excitation) to be up to 8 mA W^−1^ and 28 fold, respectively, which are comparable values with previous publications. Our hybrid 2D structure between rGO and CuO enables a potential approach for developing low-cost but high-performance optoelectronic devices, especially photodetectors, in the future.

## Abstract 2

The 2D CuO/rGO hybrid broadband photodetector is capable of stable operation in a wide light range from UV to NIR.

## Graphical abstract

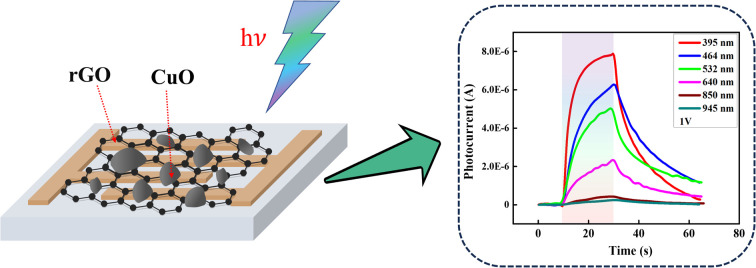

## 1. Introduction

Nowadays, manufacturers have to deal with a massive quantity of information simultaneously with a highly accurate level to keep pace with the tremendously high development rate of the industrial revolution. This demand has led to the invention of robots – a valuable assistant for humans in almost every aspect of development.^1^ At first, robots were created with the purpose of supporting people in assembly lines; however, they can perform much more complicated tasks such as manufacturing, entertainment, delivery processing, and so on.^2–4^ Regardless of the various shapes and sizes according to the practical requirements, robots are fabricated with two main components: mechanical details and programmed software.^5^ Information after being collected by robots through these parts is handled and transferred to directors.^6^

To fully detect changes in the environment, an indispensable part of robots is sensors, especially optical sensors or photodetectors.^7^ When a robot is exposed to light, these devices receive photon excitation and transform it into electrical signals, which are subsequently delivered to a processor.^8^ Based on the light operating region, optical sensors are classified into ultraviolet (UV), visible (Vis), infrared (IR), and broadband photodetectors.^9–12^ These days, one of the requirements of photodetectors used in robots is that they are sensitive to a wide range of wavelengths (*i.e.*, broadband) so that any changes in the surrounding environment can be fully detected. For this reason, scientists are carrying out numerous studies to optimize appropriate materials for broadband photodetectors with a simple and low-cost manufacture process to meet the demands of industrial sectors.^13,14^

2D materials are now attracting scientists due to their outstanding properties, such as a large surface area, which enables them to be remarkably sensitive to photon signals, tunable bandgap, and easy synthesis at low cost.^15–17^ In 2019, a black phosphorus (BP)-based photodetector fabricated by a group of Li Huang exhibited a high responsivity with a value of 23 A W^−1^ under 368 nm illumination.^18^ Moreover, while devices based on hexagonal boron nitride (h-BN) can detect deep UV light at 210 nm with an extremely low excitation power density,^19^ one fabricated from molybdenum disulfite (MoS_2_) provided sensitivity to a large band from 445 to 2717 nm with the maximum values of *R* = 50.7 mA W^−1^ and *D* = 1.55 × 10^9^ Jones.^20^ Recently, reduced graphene oxide (rGO) – a derivative of graphene – has arisen as a potential candidate for optoelectronic and photonic applications due to its unique advantages. In more detail, in the basic form, graphene possesses a 2D honeycomb structure with noticeable electrical conductivity and ultrahigh mobility which originates from the interaction between electrons and carbon atoms in the crystal lattice.^21^ More interestingly, along with its high flexibility and stability, graphene possesses excellent features such as low cost and producible through simple fabrication processes for the industrial scale.^22,23^ However, rGO-based photodetectors still face severe drawbacks, as the weak absorption and fast carrier recombination reduce devices' performance.^24,25^ Nevertheless, these challenges can be overcome by some modification techniques such as decorating and doping methods. One of the easiest ways is to combine rGO with other materials to create a hybrid structure. For instance, Peng Xiao *et al.* introduced a photodetector based on a rGO–MoS_2_/pyramid Si heterojunction, which exhibited high performance with a considerably high responsivity of 21.8 A W^−1^ and ultrabroad spectrum response from 350 to 4300 nm.^26^ Besides, in 2020, a UV-to-Vis perovskite quantum dots-rGO photodetector fabricated by a group of Farzana Chowdhury indicated a sensitivity toward a wide light range with the value of specific detectivity reaching 1 × 10^13^ Jones.^27^ By this approach, a photodetector can take advantage of constituting components and overcome the limitations of devices based on a single material. However, the materials and fabrication methods used in current research are expensive and time-consuming, limiting their potential for industrial scale-up.^28,29^ Therefore, there is a need for further research to explore and develop cost-saving and efficient methods for the fabrication of hybrid structures and to investigate the use of alternative materials with similar properties that may be more readily available and affordable.

Herein, our study investigates the potential of a low-cost 2D CuO/rGO nanohybrid which was synthesized as an active material for broadband photodetectors. In this structure, rGO with high mobility was employed as a transport layer while 2D CuO with a large surface area was employed as an “antenna material” that absorbs photon signals to generate electron–hole pairs. The 2D CuO/rGO hybrid photodetector demonstrates an exceptional capability to detect light in the Vis to near IR range with multiple wavelengths consisting of 395, 464, 532, 640, 850, and 945 nm. Specifically, the maximum photocurrent reached *ca.* 13.3 μA (under exposure to 464 nm excitation, at an intensity of 156 mW cm^−2^). Additionally, the device's responsivity exhibited the highest value of 8 mA W^−1^ (under exposure to 395 nm light at an intensity of 37 mW cm^−2^). Based on these results, we believe that our 2D CuO/rGO hybrid structure has become a potential material for practical applications and industrial fabrication.

## 2. Experimental

### 2.1 Chemical materials

The chemicals used in this experiment include: copper(ii) nitrate trihydrate (Cu(NO_3_)_2_·3H_2_O, 99% purity, Sigma Aldrich), graphene oxide nanoflakes (99% purity, Sigma Aldrich), acetone (C_3_H_6_O > 99,99% purity, Chemsol), ethanol (C_2_H_5_OH, 99,99% purity, Chemsol), and hydrazine hydrate solution (NH_2_NH_2_, 99,99% purity, Sigma Aldrich).

### 2.2 Materials fabrication process

The synthesis of the 2D CuO nanomaterial is described in Fig. S1 (ESI†). Firstly, Cu(NO_3_)_2_·H_2_O was mixed with NaOH in a beaker at a mass ratio of 1 : 10. After 1 h, the solution underwent a 4 h hydrothermal process at 180 °C. The resulting sample was rinsed and filtered multiple times before being dried at 100 °C for 6 h to collect the 2D CuO nanoplates (powder). Next, 2D CuO was dispersed in C_2_H_5_OH, forming a 2D CuO dispersion with a CuO loading of 0.5 mg mL^−1^.

The formation of the rGO charge transport layer is illustrated in Fig. S2.† Briefly, 0.5 mg mL^−1^ of GO (pre-dispersed in C_2_H_5_OH) was spray-coated onto a glass substrate. Then, the GO coated substrate was exposed to hydrazine vapor at 60 °C for 18 h to reduce the functional groups of GO, forming a rGO-coated sample. Finally, the hybrid structure of CuO and rGO was completed by spin-coating 0.5 mL of the 2D CuO dispersion (at 300 rpm for 30 s) on the as-prepared rGO layer, which was then dried at 100 °C for 1 h.

### 2.3 Device fabrication and characterization

The photodetector fabrication involved silver electrodes on a 0.7 cm^2^ channel area. The 2D CuO/rGO hybrid thin film, which was synthesized as described in the previous section, was then deposited onto the patterned electrodes to form the active layer of the photodetector. The crystal structures of rGO, 2D CuO, and 2D CuO/rGO were evaluated by X-ray diffraction (XRD) using a D8 Advance-Bruker diffractometer which was operated at 40 kV and 100 mA and a Cu/Kα radiation source (*λ* = 0.154 nm), a high-resolution transmission electron microscope (HR – TEM, JEM – 2100 Plus, JEOL) with an accelerating voltage of 200 kV equipped with a LaB6 filament and an energy dispersive X-ray (EDS) spectrometer (Ultim Max TEM, JEOL). The optical properties were recorded using an ultraviolet-visible (UV-vis) spectrophotometer (JASCO V670). Raman spectra were recorded with a Raman spectrometer (Xplora One, HORIBA) with a 532 nm excitation source, a capacity of 5 mW, a 10× objective lens, and an acquisition time of 15 s for each spectrum. The surface morphology was observed with a scanning electron microscope (SEM, Hitachi S-4800). Photodetector characteristics were investigated through *I*–*V* and *I*–*t* curves using a Keithley 2400 system. The light sources used for I–V and *I*–*t* measurements include 395 nm (model M395L4 LED from THORLABS), 464 nm (model COBW1-480 from TOPAI), 532 nm and 640 nm (RG43A1 from THORLABS), 850 nm (model M850L3 from THORLABS) and 945 nm (model JH-5050IR12G42-T8A-940 from LEDGUHON).

## 3. Results and discussion

### 3.1 Fabrication and characterization

**Fig. 1:**
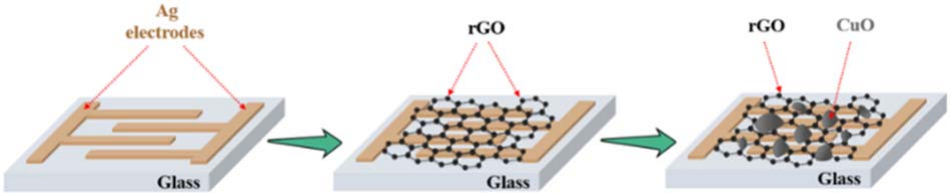
Schematic fabrication process of the 2D CuO/rGO hybrid photodetector.

The fabrication procedure of the devices is depicted in Fig. 1. After the deposition of Ag electrodes, 2D CuO/rGO active materials were developed onto the substrate to complete the hybrid photodetector (see more details in the Experimental section).

**Fig. 2:**
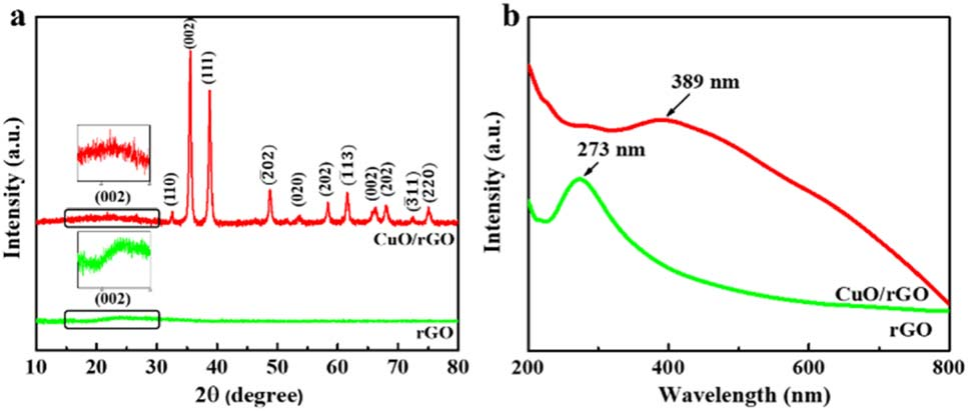
(a) XRD patterns and (b) UV-vis spectra of rGO and hybrid 2D CuO/rGO samples.

Before evaluating the performance of a device, the properties of the active material layer need to be studied. The XRD patterns of rGO and 2D CuO/rGO samples are presented in Fig. 2a. In the hybrid sample, a characteristic diffraction peak of rGO was observed at 24.4°. Additionally, other peaks were determined at 33.6°, 35.4°, 38.5°, 48.7°, 54.2°, 58.3°, 61.5°, 66.3, 68.2°, 73.2° and 75.1° representing the (110), (002), (111), (2̄02), (020), (202), (1̄13), (002), (202), (3̄11) and (220) planes of the monoclinic phase of 2D CuO. These results match well with the XRD data of bare 2D CuO shown in Fig. S3a† and in previous reports;^30,31^ therefore, the presence of both 2D CuO and rGO in the hybrid sample is confirmed. Otherwise, no exotic peak was observed in the XRD patterns. In general, the sharp and narrow diffraction peaks indicate that 2D CuO has good crystallinity and no strange phase appeared during the experiments.

UV-vis absorption spectra were applied to determine the optical characteristics of rGO and the 2D CuO/rGO hybrid, and the results are shown in Fig. 2b. Clearly, it is shown that rGO has an absorption peak at 273 nm for removing functional groups from the GO structure.^32^ Moreover, as indicated in Fig. S3b,† there is an absorption peak at around 360 nm, which is consistent with the excitonic absorption of 2D CuO.^33^ These results are consistent with some studies reported previously by the group of Sadur Rifki and the group of Anupamjeet Kaur.^34,35^ When rGO is combined with 2D CuO, the maximum absorption peak shifts to 389 nm, which can be explained by forming a Cu–O–C bond after the synthesis process.^36^

**Fig. 3:**
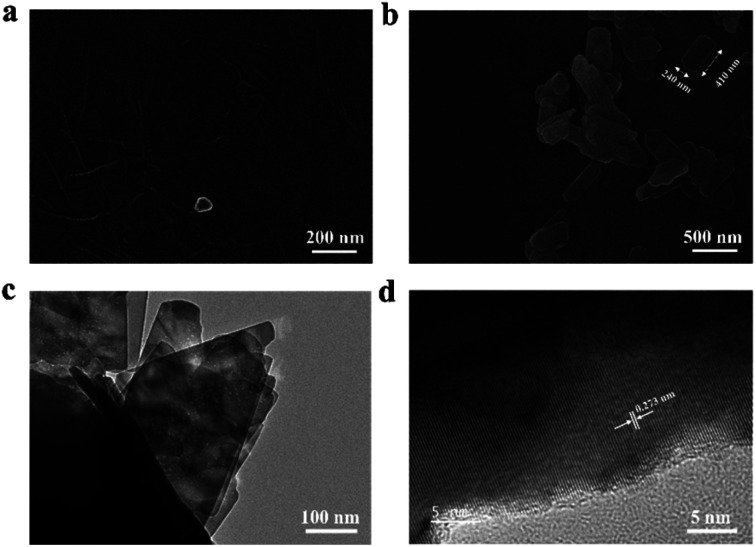
(a and b) SEM images of a rGO nanosheet at the 200 nm scale and CuO/rGO at the 500 μm scale, respectively; (c and d) TEM image and HRTEM image of 2D CuO/rGO, respectively.

The morphological properties of the as-synthesized materials are depicted by SEM images in Fig. 3. Thin-paper rGO sheets are formed after the hydrazine reduction process (Fig. 3a).^37^ This formation is further confirmed by rGO Raman spectra in Fig. S4,† where the signals of in-phase vibration of the graphite lattice (G-band) at 1575 cm^−1^ and the disorder band originating from graphite edges (D-band) at about 1385 cm^−1^ are observed.^38^ In Fig. 3b and c, uniform 2D CuO nanoplates appear like discrete clusters on the rGO sheets. From the analysis of the SEM image (Fig. S5†), the average length and width of these nanoplates were estimated to be *c.a.* 410 nm and 240 nm, respectively. In our study, since CuO was coated onto rGO by a physical spray-coating technique, van der Waals interaction plays an important role in the networking of 2D nanoplates and the rGO layer. The HRTEM image in Fig. 3d shows a cluster of 2D CuO. Here, the distance between two neighboring fringes of 2D CuO was calculated to be 0.273 nm, and the SAED pattern in Fig. S6† (obtained using TEM equipment) exhibited bright dots attributed to the (110) crystal plane.^39^ Therefore, the results confirmed the well crystalline structure formed with almost no defects in 2D CuO, and the results are consistent with the XRD analysis.

**Fig. 4:**
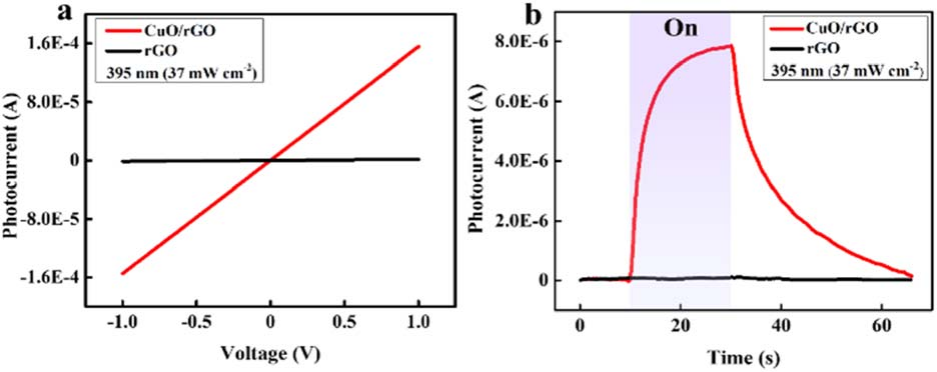
(a) *I*–*V* characteristics and (b) *I*–*t* measurements of rGO and 2D CuO/rGO photodetectors under exposure to 395 nm light.

Photodetector's characteristics are investigated using current–voltage (*I*–*V*) and current depending on time (*I*–*t*) curves under a fixed bias of 1 V. In Fig. 4a, both rGO and 2D CuO/rGO-based devices exhibit linear *I*–*V* relations, demonstrating good ohmic contacts at the 395 nm wavelength. However, the photodetector based on bare rGO shows poor light sensitivity because the photocurrent was negligible. In contrast, in Fig. 4b, the photocurrent through the device rapidly rose when the 395 nm light was turned on and decreased after turning of the light. The same behavior is observed when the devices are illuminated with the 464 nm wavelength, as shown in Fig. S7.† The photocurrent (*I*_ph_) of a photodetector is calculated using the formula: *I*_ph_ = *I*_light_ − *I*_dark_,^40^ in which, *I*_dark_ is the current measured in the dark, and *I*_light_ is the current recorded 20 s after turning on the light. In Fig. S6,† the *I*_ph_ values of the 2D CuO/rGO device under 395 and 464 nm light sources were calculated to be 7.8 μA and 13.3 μA, respectively. In our report, the response time of a photodetector was defined as the time to reach 90% of its maximum photocurrent, while the recovery time was determined as the duration for the photocurrent to return to 10% of its highest value.^41^ As presented in Fig. S8a and b,† the response and recovery times of the 2D CuO/rGO hybrid device under exposure to 395 nm light are 8.2 s and 24.8 s, respectively, while those under the 464 nm light source are 9.6 s and 25.7 s, respectively.

**Fig. 5:**
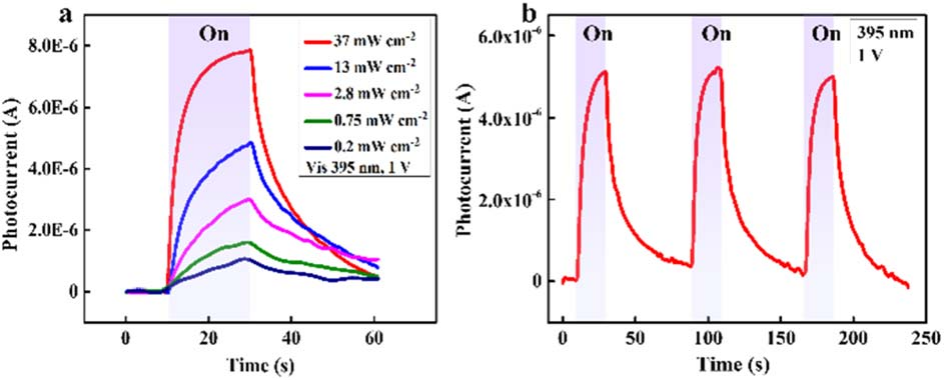
(a) *I*–*t* curves of our hybrid photodetector toward different intensities of 395 nm light and (b) response repeatability in three cycles of exposure.

Fig. 5a displays the *I*–*t* curves of the device under a 395 nm light source with various intensities. When the light intensity increased from 0.2 mW cm^−2^ to 37 mW cm^−2^, the photocurrent rose from 1.6 μA to 7.8 μA. These accelerations can be explained by the photocurrent and light intensity relationship: *I*_ph_ = *AP*^*θ*^, where *A* represents the wavelength constant, and *θ* stands for the exponential. Therefore, *I*_ph_ is proportional to the intensity *P.*^42^

The stability of the hybrid photodetector was estimated by continuously turning on and off the LED of 395 nm wavelength, and the result is presented in Fig. 5b. There is no considerable change in photocurrent after several measuring cycles, which means that the device is suitable for long-term operation. Fig. S9† shows similar characteristics of the device under the 464 nm wavelength. Indeed, when the excitation intensity increased, the photocurrent went up accordingly (Fig. S9a†), and there was almost no noticeable reduction in the device's performance after continuous investigation cycles. Interestingly, these behaviors are also observed under multiple wavelengths, comprising 532 nm, 640 nm, 850 nm, and 945 nm, as shown in Fig. S10.†

On the other hand, three other parameters including responsivity (*R*), photoconductive gain (*G*) and detectivity (*D*) were calculated to assess the photodetector's performance. First, responsivity demonstrates the capability to convert an optical signal to an electrical signal of a photodetector, which can be presented by eqn (1):1
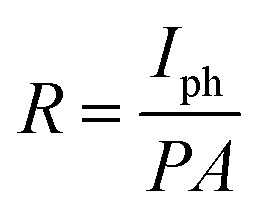
Then, photoconductive gain, which is defined as the number of carriers detected per absorbed photon, can be expressed by eqn (2):2
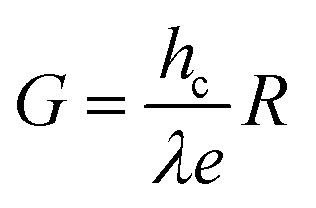
And finally, the ability of a photodetector to detect weak signals of light, known as detectivity, was obtained from eqn (3):3
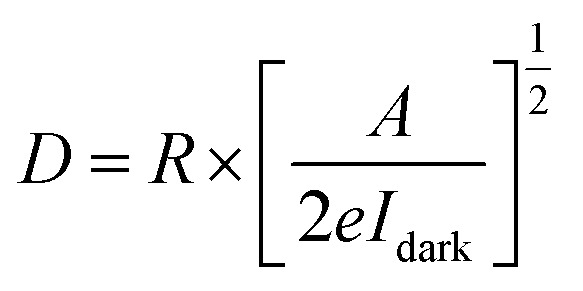
In these equations, *I*_ph_ stands for the photocurrent, *P* and *A* are the light intensity and the effective area of the device (0.7 cm^2^), *h* and *c* represents Planck's constant and light's velocity, and *λ* and *e* are the wavelength and the electron charge, in the given order.^43–45^ Interestingly, the calculated *R* and *G* of our device matched well with the theory: 
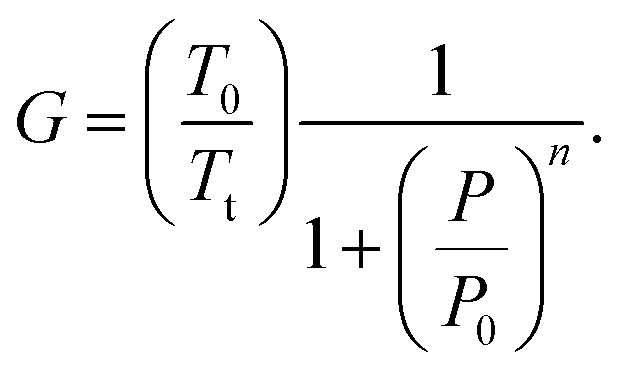
 Here, *P*_0_ is the excitation intensity where the surface states are fully filled, *T*_0_ is the carrier life-time at considerably low excitation intensity (*P* → 0), *T*_t_ is the carrier transit time, and *n* is a phenomenological fitting parameter (*n* ≈ 1).

**Fig. 6:**
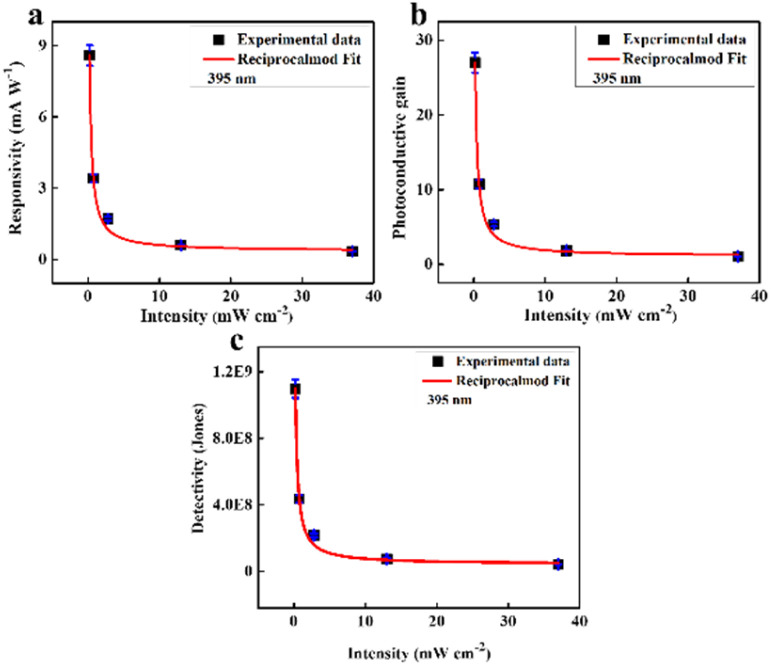
(a) Responsivity, (b) photoconductive gain and (c) detectivity of the 2D CuO/rGO device including reciprocal function fitting lines with error bars.

**Fig. 7:**
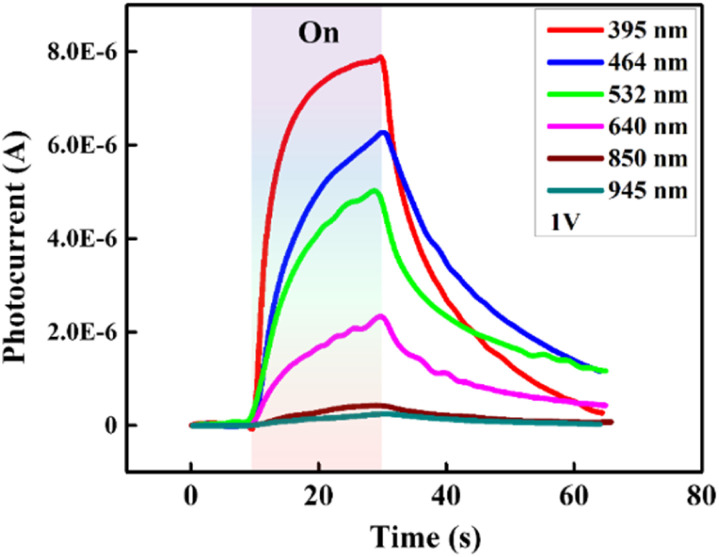
Time-dependent current measurement of the hybrid photodetector under six lighting sources with specific wavelengths of 395, 464, 532, 640, 850, and 945 nm.

Moreover, the maximum value of *R* and *G* could be estimated from curve fitting to the reciprocal function: 
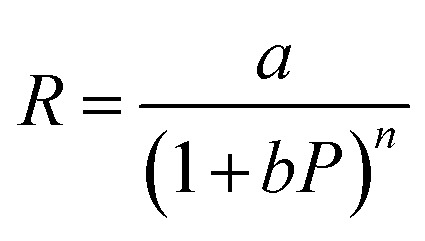
 and 
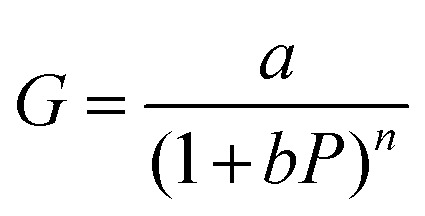
, in which *a* and *b* are constants. Both *R* and *G* decrease with the increase of *P*, and in Fig. 6, the highest values of *R* and *G* are recorded from the fitted curves, which are 8.8 mA W^−1^ and 28 fold under the 395 nm light, while under the 464 nm light, those values are 5.3 mAW^−1^ and 14 fold, respectively (please see Fig. S11†). The increase in photocurrent at all wavelengths confirms that our hybrid device possesses broadband photodetection capability, consistent with the UV-vis absorption results mentioned above in Fig. 7.

The summary of our photodetector results and comparison with some other reported devices are presented in Table 1. Although the performance of this hybrid 2D CuO/rGO device is not superior, it is acceptable as compared to the results from previous publications.

**Table 1:** Comparison of the responsivities of other rGO photodetectors

Active materials	Wavelength *λ* (nm)	Responsivity *R* (mA W^−1^)	Ref.
*p*-Phenylendiamine-rGO/Si	382	1.4	46
rGO/GaN	382	1.54	47
WS_2_-QDs/rGO	405	5.22	48
rGO-SiNW	532	0.33	49
rGO-(Cd : Zn)S	470	9.2	50
rGO films	530	2.4	51
Hybrid 2D CuO/rGO	395	8.8	This work
	464	5.3	

### 3.2 Sensing mechanism

**Fig. 8:**
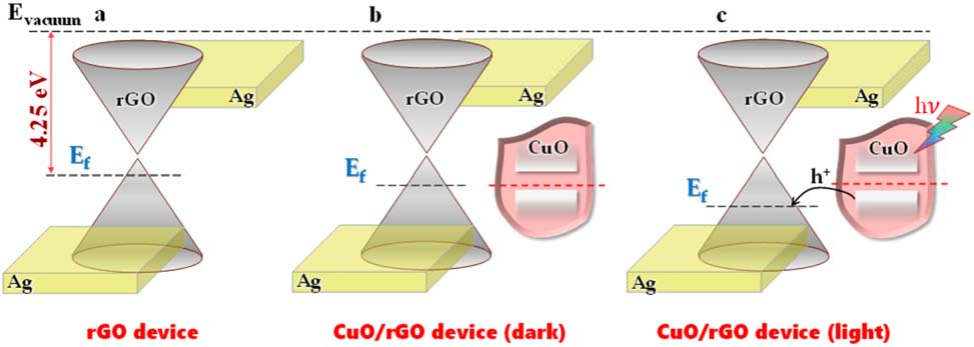
The proposed sensing mechanism of rGO and hybrid CuO/rGO photodetectors. Energy diagram of (a) the rGO device and the hybrid device under (b) dark conditions and (c) light condition.

Regarding the charge transfer mechanism, in this hybrid structure, rGO worked as the hole-transporting layer while 2D CuO was the main material absorbing visible light for charge carrier generation. Fig. 8 illustrates the charge transfer direction between rGO and CuO. As reported in other studies, the work function of rGO is about 4.25 eV,^52,53^ and that of 2D CuO is around 4.70 eV.^54,55^ When two materials were combined, their Fermi levels aligned until reaching equilibrium, and a hole accumulation layer was formed at the CuO–rGO interface. On the other hand, since CuO surfaces could adsorb oxygen molecules, the hole concentration in the rGO layer slightly increased. Under light exposure, electron–hole pairs were generated inside 2D CuO. Because the valence band of rGO is higher than that of 2D CuO, the photo-excited holes easily transferred into rGO. Under an external electric field, electrons and holes inside rGO can be driven toward the silver electrodes leading to photocurrent enhancement.

## 4. Conclusion

In summary, we have successfully fabricated a low-cost photodetector based on a 2D CuO/rGO hybrid structure through a simple solution route, which exhibits a fast and stable response to vis and NIR. Furthermore, the 2D CuO/rGO device was also compared with a pristine rGO-based device to clarify the contribution of CuO to the sensitivity of the hybrid structure toward incident light and the results are thoroughly presented. Under 395 nm light illumination, the device's response and recovery time were respectively 8.2 s and 24.8 s, and the photocurrent was about 7.8 μA at *P* = 37 mW cm^−2^. Meanwhile, under 464 nm light illumination, those values were 10.6 s, 38.7 s and 13.3 μA at *P* = 156 mW cm^−2^. The maximum responsivity was 8.8 mA W^−1^ and 5.3 mA W^−1^ under 395 nm and 464 nm light illumination, respectively. Furthermore, with the recorded sensitivity toward longer wavelengths (532, 640, 850 and 945 nm), we do believe that our 2D CuO/rGO hybrid structure is a promising structure for low-cost processed broadband PDs in future.

## Author contributions

D. A. Ngo: conceptualization, methodology, format analysis, investigation, and writing – original draft; N.M. Nguyen: data curation and methodology; C. K. Tran: format analysis and software; T. N. H. Tran: investigation and writing – review & editing; L.T. Duy: resources, software, and writing – review & editing; T. T. T. Bui: validation and data curation; T. T. V. Tran: visualization and data curation; V. Q. Dang: funding acquisition, supervision, project administration, and writing – review & editing.

## Conflicts of interest

There are no conflicts to declare.

## Supplementary Material

NA-006-D3NA00796K-s001
