# Supplementary material for: A study on a broadband photodetector based on hybrid 2D copper oxide/reduced graphene oxide
Source: Nanoscale Adv. 2024 Jan 25;6(5):1460–6. doi: 10.1039/d3na00796k (PMC10898423; doi:10.1039/d3na00796k)
Supplement: NA-006-D3NA00796K-s001 [file NA-006-D3NA00796K-s001.pdf]

Electronic Supplementary Material (ESI) for Nanoscale Advances.
This journal is © The Royal Society of Chemistry 2024

A STUDY ON BROADBAND PHOTODETECTOR BASED ON HYBRID 2D COPPER OXIDE/ REDUCED GRAPHENE OXIDE

Duc Anh Ngo[a,c], Nhat Minh Nguyen[a,c], Cong Khanh Tran[a,c], Thi Thanh Van Tran[a,c], Nhu Hoa Thi Tran [a,b,c], Thi Thu Thao Bui[a,c], Le Thai Duy[a,c], Vinh Quang Dang*[a,b,c].

[a] Faculty of Materials Science and Technology, University of Science, 227 Nguyen Van Cu Street, District 5, Ho Chi Minh City 700000, Vietnam
[b] Center for Innovative Materials and Architectures (INOMAR), Ho Chi Minh City 700000, Vietnam
[c] Vietnam National University, Ho Chi Minh City (VNU-HCM) 700000, Vietnam

* Correspondence. E-mail: *vinhquangntmk@gmail.com*

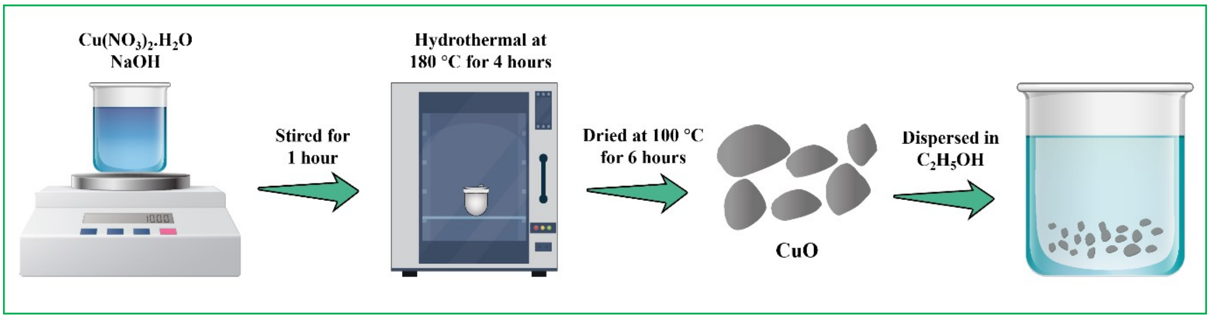

Figure S1. *Synthesis process of 2D CuO dispersion.*

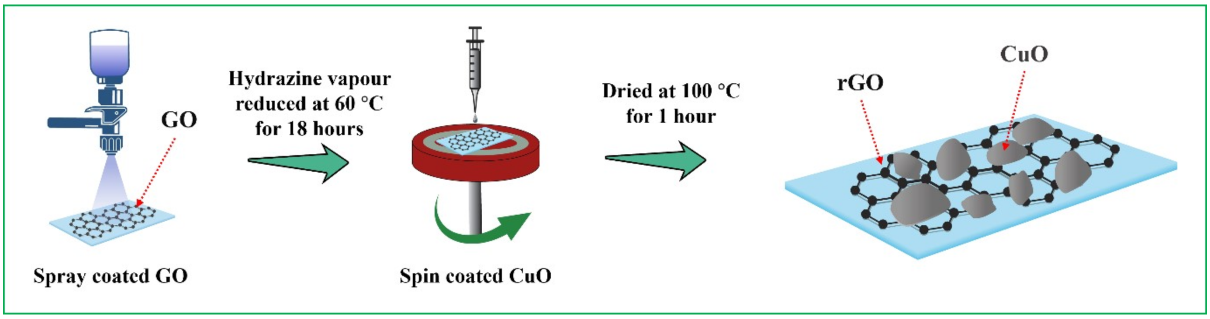

Figure S2. *Synthesis process of 2D CuO/rGO hybrid thin film.*

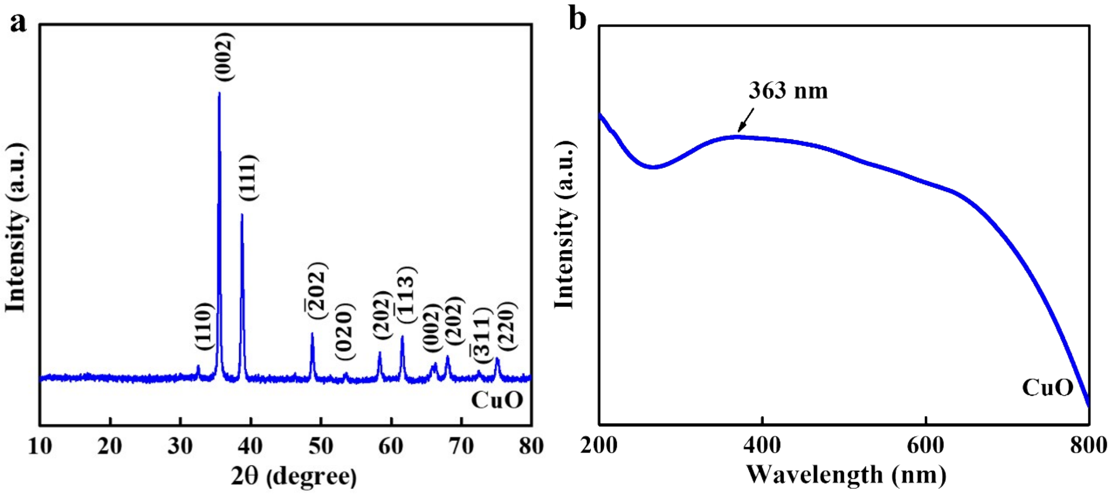

Figure S3. *(a) XRD pattern and (b) UV-vis spectrum of 2D CuO sample*

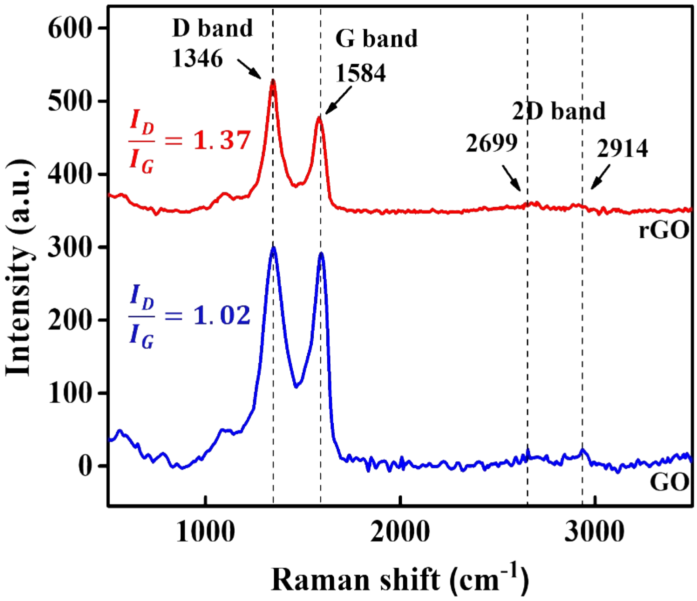

Figure S4. *Raman spectra of GO and rGO samples*

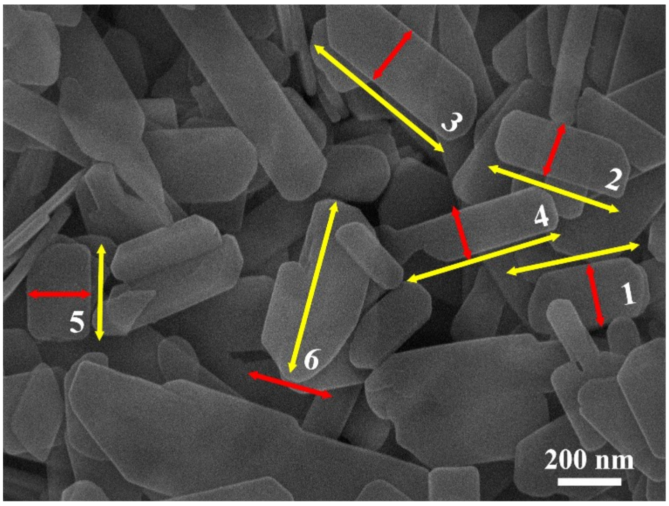

No.	Length (nm)	Width (nm)
1	406	236
2	412	232
3	423	243
4	414	231
5	369	234
6	432	252
Average	~ 410	~ 240

Figure S5. *SEM image and size statistics of 2D CuO nanoplates*

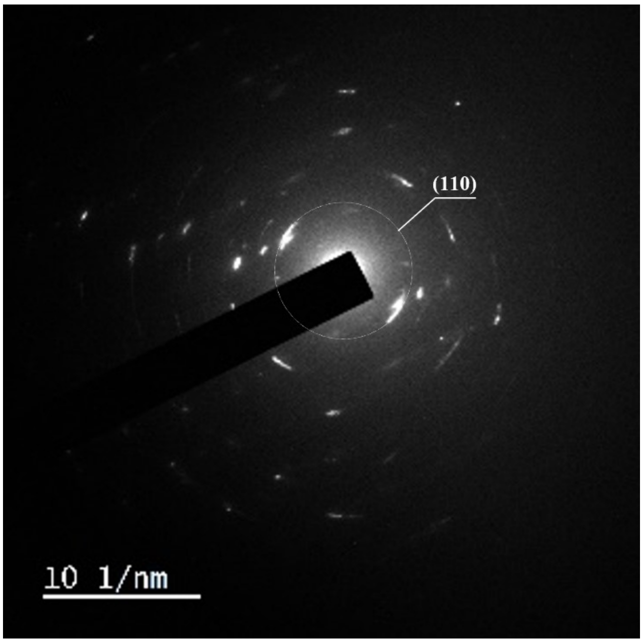

Figure S6. *SAED pattern of 2D CuO/rGO*

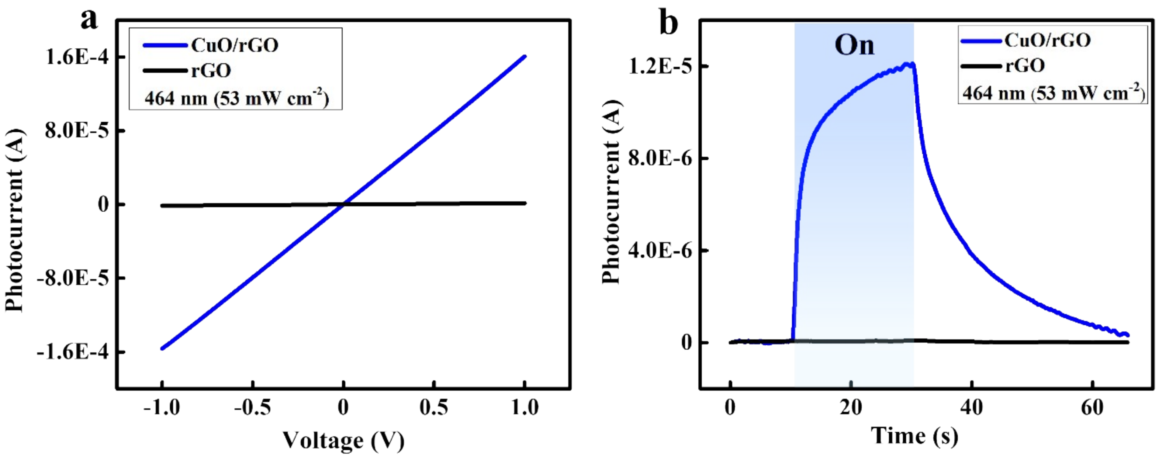

Figure S7. *(a) I–V and (b) I-t characteristics of rGO and 2D CuO/rGO photodetectors under 464 nm light*

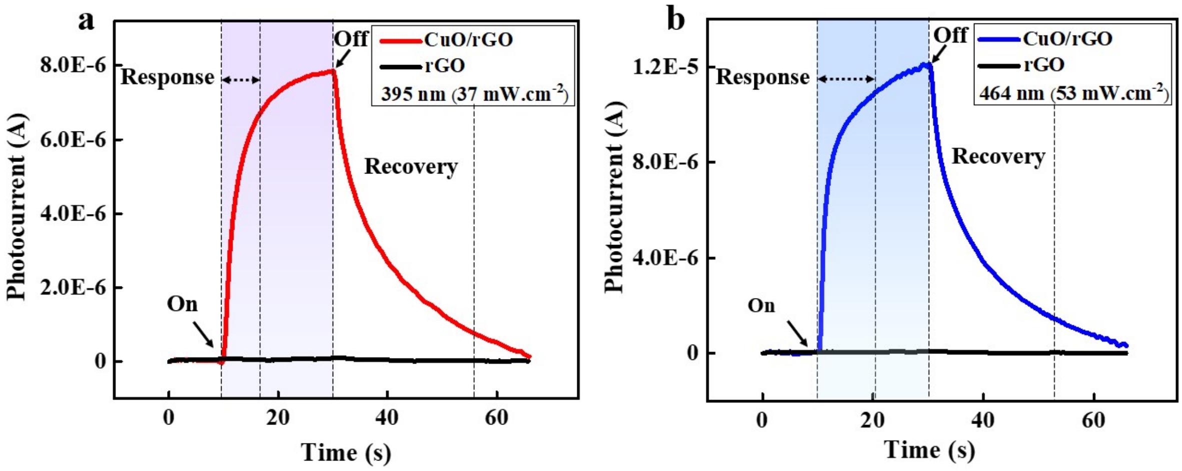

Figure S8. *Response and recovery time of a hybrid photodetector under exposure to (a) 395 nm and (b) 464 nm lights*

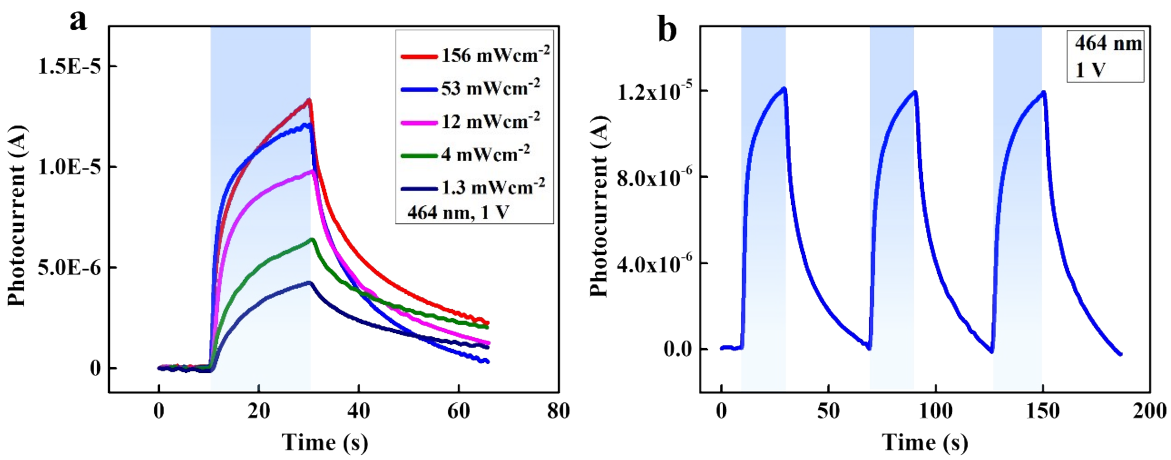

Figure S9. *(a) I–t curves and (b) the repeatability of a hybrid photodetector under 464 nm light*

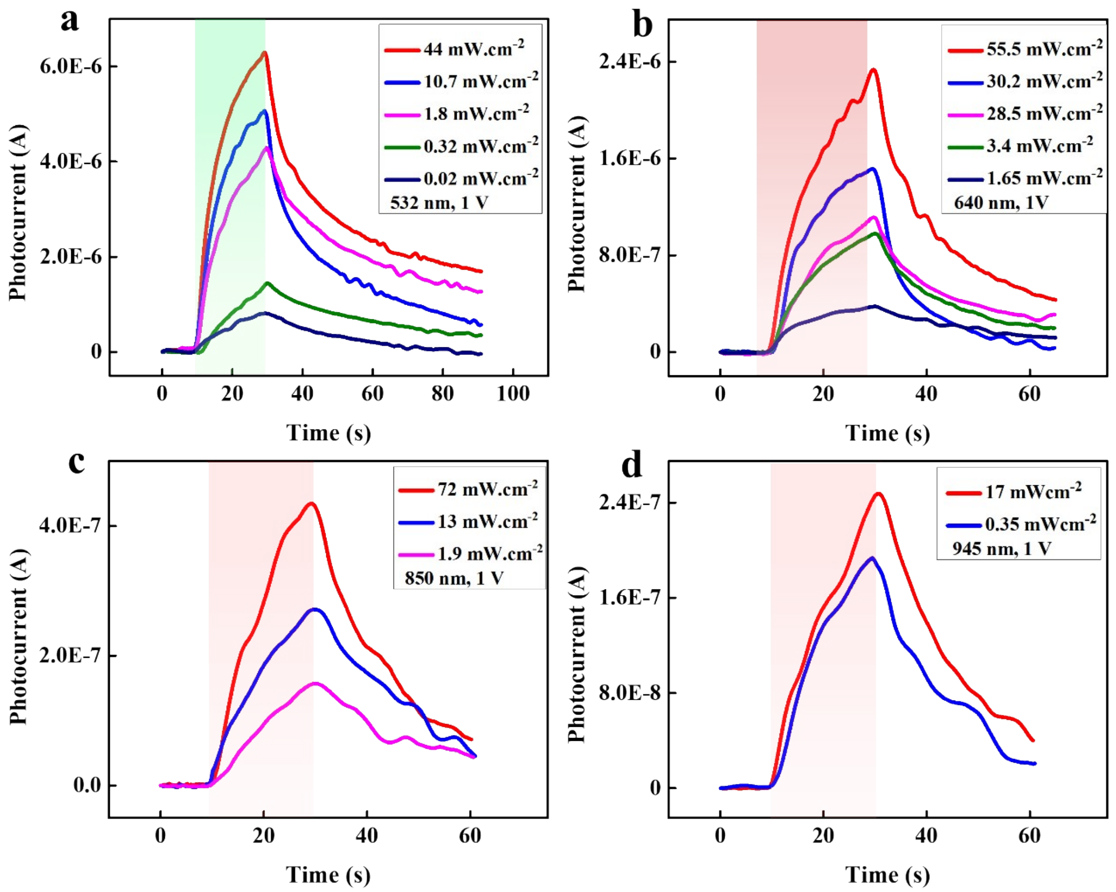

Figure S10. *I–t curves of the hybrid device under exposure to light at wavelengths of (a) 532 nm, (b) 640 nm, (c) 850 nm, and (d) 945 nm.*

The hybrid device generated the highest photocurrents of 6.3, 2.3, 0.43, and 0.25 μA when excited by the 532-, 640-, 850-, and 945-nm wavelengths, respectively. When the light was ON, the photocurrent immediately increased, and when the light was OFF, the photocurrent decreased. Notably, it gradually decreased as increasing the light intensity.

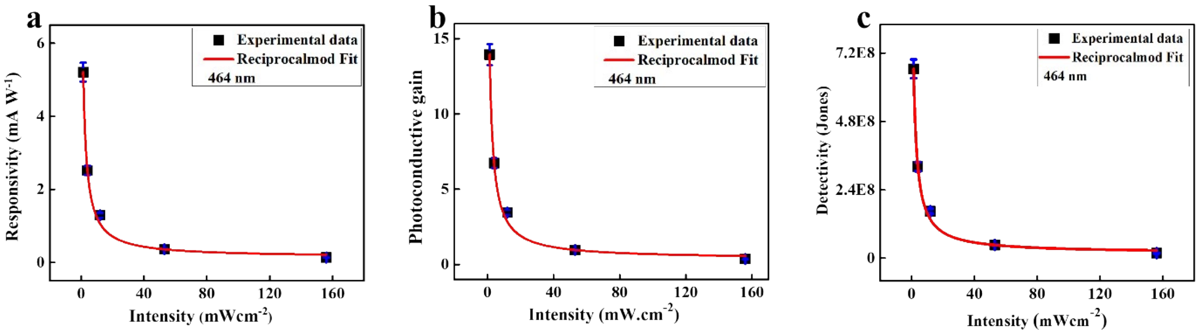

Figure S11. *(a) Responsivity, (b) photoconductive gain and (c) detectivity of 2D CuO/rGO device including reciprocal function fitting lines with error bar.*
